# Treatment of giant cell arteritis with ultra-short glucocorticoids and tocilizumab: the role of imaging in a prospective observational study

**DOI:** 10.1093/rheumatology/kead215

**Published:** 2023-05-17

**Authors:** Francesco Muratore, Chiara Marvisi, Giulia Cassone, Luigi Boiardi, Pamela Mancuso, Giulia Besutti, Lucia Spaggiari, Massimiliano Casali, Stefania Croci, Annibale Versari, Paolo Giorgi Rossi, Mariagrazia Catanoso, Massimo Costantini, Elena Galli, Carlo Salvarani

**Affiliations:** Rheumatology Unit, Azienda Unità Sanitaria Locale-IRCCS di Reggio Emilia, Reggio Emilia, Italy; University of Modena and Reggio Emilia, Modena, Italy; Rheumatology Unit, Azienda Unità Sanitaria Locale-IRCCS di Reggio Emilia, Reggio Emilia, Italy; University of Modena and Reggio Emilia, Modena, Italy; Rheumatology Unit, Azienda Ospedaliero-Universitaria di Modena, Modena, Italy; Rheumatology Unit, Azienda Unità Sanitaria Locale-IRCCS di Reggio Emilia, Reggio Emilia, Italy; Epidemiology Unit, Azienda Unità Sanitaria Locale-IRCCS di Reggio Emilia, Reggio Emilia, Italy; University of Modena and Reggio Emilia, Modena, Italy; Radiology Unit, Azienda Unità Sanitaria Locale-IRCCS di Reggio Emilia, Reggio Emilia, Italy; Radiology Unit, Azienda Unità Sanitaria Locale-IRCCS di Reggio Emilia, Reggio Emilia, Italy; Nuclear Medicine Unit, Azienda Unità Sanitaria Locale di Piacenza, Piacenza, Italy; Clinical Immunology, Allergy and Advanced Biotechnologies Unit, Azienda Unità Sanitaria Locale-IRCCS di Reggio Emilia, Reggio Emilia, Italy; Nuclear Medicine Unit, Azienda Unità Sanitaria Locale-IRCCS di Reggio Emilia, Reggio Emilia, Italy; Epidemiology Unit, Azienda Unità Sanitaria Locale-IRCCS di Reggio Emilia, Reggio Emilia, Italy; Rheumatology Unit, Azienda Unità Sanitaria Locale-IRCCS di Reggio Emilia, Reggio Emilia, Italy; Scientific Directorate, Fondazione IRCCS Istituto Nazionale dei Tumori di Milano, Milano, Italy; Rheumatology Unit, Azienda Unità Sanitaria Locale-IRCCS di Reggio Emilia, Reggio Emilia, Italy; University of Modena and Reggio Emilia, Modena, Italy; Rheumatology Unit, Azienda Unità Sanitaria Locale-IRCCS di Reggio Emilia, Reggio Emilia, Italy; University of Modena and Reggio Emilia, Modena, Italy

**Keywords:** tocilizumab, treatment, PET/CT, GCA, large-vessel vasculitis

## Abstract

**Objectives:**

To assess the impact of tocilizumab (TCZ) monotherapy after ultra-short-pulse glucocorticoids (GCs) on clinical manifestations, and vessel inflammation and damage in large vessel-GCA (LV-GCA).

**Methods:**

In this prospective observational study, we enrolled patients with active LV-GCA. All patients received 500 mg per day i.v. methylprednisolone for three consecutive days and weekly s.c. TCZ injections from day 4 until week 52. PET/CT was performed on all patients at baseline and at weeks 24 and 52. The primary end points were the reduction in the PET vascular activity score (PETVAS) at weeks 24 and 52 compared with baseline, and the proportion of patients with relapse-free remission at weeks 24 and 52. The secondary end point was the proportion of patients with new aortic dilation at weeks 24 and 52.

**Results:**

A total of 18 patients were included (72% female, mean age 68.5 years). Compared with the baseline value, a significant reduction in the PETVAS was observed at weeks 24 and 52, mean (95% CI) reductions –8.6 (–11.5 to –5.7) and –10.4 (–13.6 to –7.2), *P* = 0.001 and 0.002, respectively. The proportion of patients with relapse-free remission at weeks 24 and 52 was 10/18 (56%, 95% CI 31–78) and 8/17 (47%, 95% CI 23–72), respectively. At weeks 24 and 52, no patient had shown new aortic dilation. However, 4 patients who had shown aortic dilation at baseline showed a significant increase in aortic diameter (≥5 mm) at week 52.

**Conclusion:**

TCZ monotherapy after ultra-short-pulse GCs controlled the clinical symptoms of GCA and reduced vascular inflammation.

**Trial registration:**

ClinicalTrials.gov, https://clinicaltrials.gov, NCT05394909.

Rheumatology key messages

- Tocilizumab monotherapy was effective in reducing vascular inflammation, as evaluated by PET/CT.
- Larger clinical trials are needed to confirm these results and to evaluate whether tocilizumab prevents vascular damage.

## Introduction

GCA is a vasculitis that involves the large- and medium-sized arteries, producing a wide spectrum of clinical manifestations [1, 2]. ^18^F-fluorodeoxyglucose (^18^F-FDG) PET combined with CT (PET/CT) can be used to assess large-vessel inflammation and is highly sensitive and specific for detecting extra-cranial GCA [3, 4]. The most feared early complication of GCA is visual loss, while aortic aneurysm is generally a late complication [1, 5]. Preliminary results suggest that patients with early aortic ^18^F-FDG uptake are at higher risk of developing aortic complications [6–9].

Glucocorticoids (GCs) are still the mainstay therapy of GCA; however, they are associated with important side effects [10]. Two randomized controlled trials (RCTs) in GCA have shown that tocilizumab (TCZ), an IL-6 receptor inhibitor, is safe and effective in reducing disease flares and has a powerful GC-sparing effect [11, 12]. However, in these two trials, the patients were concomitantly treated with high-dose GCs for at least 26 weeks. There are preliminary data suggesting that TCZ in monotherapy without GCs or after ultra-short-pulse GC administration is able to induce and maintain remission, and may reduce ultrasonographic evidence of temporal artery inflammation in patients with newly diagnosed GCA [13–15]. However, it is unknown whether TCZ alone or in association with GC can resolve the vessel inflammation and prevent the development of aortic complications.

We conducted this observational study, Treatment Of giant cell arteritis Patients with ultra-short glucocorticoids And tociliZumab: the role of Imaging in a prospective Observational study (TOPAZIO), to assess the impact of TCZ monotherapy after ultra-short pulses of GC administration on clinical manifestations, and vessel inflammation and damage in active GCA with evidence of large-vessel involvement (LV-GCA).

## Methods

### Study design and participants

This prospective observational study enrolled consecutive patients aged more than 50 years with active, newly diagnosed or relapsing LV-GCA at the Department of Rheumatology, Azienda USL-IRCCS di Reggio Emilia, Italy. LV-GCA was defined by the presence of large-vessel inflammation on PET/CT, with or without cranial manifestations and pathological or US evidence of temporal artery involvement.

The study aims were:

- to evaluate the functional and morphological imaging changes after 24 and 52 weeks of TCZ monotherapy preceded by ultra-short GC pulses compared with baseline values.
- to evaluate the proportion of patients with relapse-free remission at weeks 24 and 52.

We included patients with active newly diagnosed or relapsing LV-GCA according to the following inclusion criteria:

- age ≥50 years
- PET/CT showing FDG uptake of ≥2 in at least one large artery (aorta and its major branches) and considered consistent with active vasculitis by the evaluation of a nuclear medicine physician (MC) with long-term expertise in vasculitis
- at least one of (1) ESR >40 mm/h or CRP >10 mg/l, and (2) cranial or systemic symptoms of GCA or symptoms of PMR.

Patients with a history of, or presenting with ischaemic cranial manifestations (jaw claudication, vision loss, amaurosis fugax, diplopia, stroke, or transient ischaemic attacks) were excluded to minimize the occurrence of disease-related severe ischaemic events. Other cranial manifestations were not considered exclusion criteria.

Other exclusion criteria included treatment with >10 mg/day of prednisone (or equivalent) for >10 consecutive days in the previous 3 months and previous treatment with TCZ. The detailed inclusion and exclusion criteria are reported in the supplementary material available at *Rheumatology* online.

### End points

The two primary end points were the variation in PET vascular activity score (PETVAS) at weeks 24 and 52 compared with baseline and the proportion of patients with relapse-free remission at weeks 24 and 52.

The secondary end points were the following:

1. Proportions of patients with new aortic dilation at weeks 24 and 52.
2. Proportions of patients with relapse-free remission at weeks 24 and 52 according to the following definitions:
  - clinical remission.
  - EULAR consensus definitions for remission in GCA and other types of large-vessel vasculitis (LVV) [16].

### Treatment

Patients received 3 boluses of 500 mg of i.v. methylprednisolone on days 1, 2 and 3 in 250 ml saline solution. Thereafter, GC treatment was discontinued, and the patients received weekly s.c. TCZ injections (162 mg) from day 4 until week 52.

### Assessment

Disease assessment was performed at each visit on days 1, 4 and 31 and every 12 weeks thereafter. In case of relapse, persistence, or worsening of GCA or PMR symptoms, GC treatment was started at any time on investigator discretion based on the severity of the manifestations.

PET/CT was performed in all patients at baseline and at weeks 24 and 52. Scans were evaluated by one nuclear medicine specialist, who was aware of the scans order but not of the patients’ clinical status, using the visual 0–3 vascular to liver FDG uptake grading scale [17]. Scans showing grade 2 and 3 FDG uptake were classified as active. Additionally, the PETVAS was calculated [18]. Detailed information on PET/CT acquisition are reported in the supplementary material available at *Rheumatology* online.

All PET/CT scans were also independently evaluated by a radiologist (non-contrast enhanced CT study), who measured the diameter of the aorta in a transverse plane at four different levels. Aortic dilation was defined by a diameter of >40 mm in the ascending aorta, of ≥40 mm in the thoracic descending aorta, and ≥30 mm in the supra and infrarenal abdominal aorta [8]. Any change of ≥5 mm on serial CT was considered significant progression of aortic damage (end point of interest) [19].

Remission was defined by all the following: absence of any clinical signs and symptoms directly attributable to GCA; normalization of CRP and ESR values; absence of new/worsened aortic damage at CT; vascular FDG uptake of <2 in all large arteries at PET/CT, or overall PET image interpretation of non-active vasculitis by the nuclear medicine physician.

Relapse was determined by the investigator and defined as one or more of the following: recurrence of signs or symptoms of GCA or PMR; CRP values of >10 mg/l, or ESR values of >40 mm/h if these were considered by the investigator to be due to GCA; evidence of worsening vascular FDG uptake at PET/CT. The definition of relapse included the start of GC therapy.

To compare the results of our study with those of other studies, we also considered in the secondary end points another two different definitions of remission:

1. clinical remission: absence of any clinical signs and symptoms directly attributable to GCA, including normalization of CRP and ESR, independently by imaging evaluation.
2. EULAR consensus definitions for remission in GCA and other types of LVV: absence of all clinical signs and symptoms attributable to active LVV, normalization of ESR and CRP values, and no evidence of progressive aortic damage at CT [16].

### Statistical analysis

The PETVAS at weeks 24 and 52 was compared with the baseline value using the Wilcoxon test for paired samples. Because of the small sample size, the exact *P*-value was calculated and adjusted for multiple testing by using the Bonferroni correction (*P*-values < 0.025 were considered significant). Analyses only included patients who completed the assessment at each time point.

The binary primary (proportion of patients with relapse-free remission at weeks 24 and 52) and secondary outcome are presented as percentages with 95% binomial exact (Clopper-Pearson) confidence intervals (95% CIs). Patients who discontinued the study before or at week 20 were imputed as non-responders to treatment.

The study protocol was approved by the Reggio Emilia Provincial Ethics Committee (0176 – 15/05/2019) and registered in ClinicalTrials.gov (NCT05394909). All patients or their relatives provided written informed consent prior to enrolment. The study was conducted in accordance with the Declaration of Helsinki.

## Results

**Figure 1.**
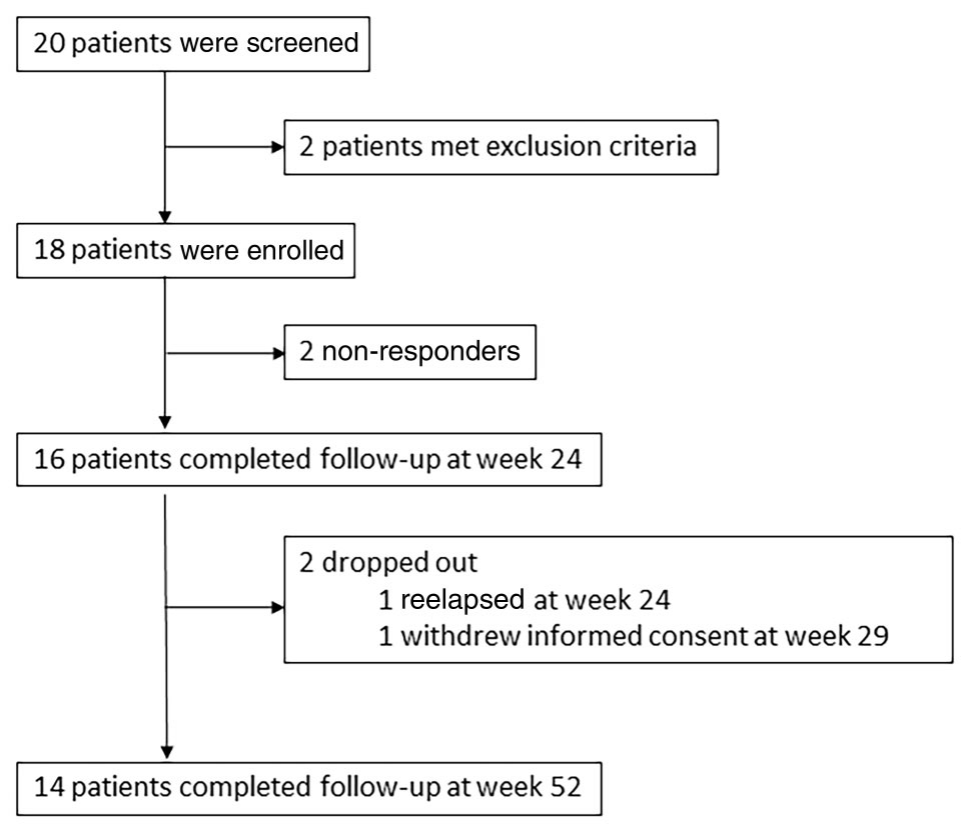
Study profile

From March 2019 to November 2020, 20 patients were screened for eligibility, and 18 patients were included (two patients met the exclusion criteria) (Fig. 1). Nine patients (50%) had newly diagnosed LV-GCA, and the remaining 9 (50%) had relapsing disease. The demographic and baseline characteristics of the study cohort are reported in Table 1. Fifteen of the 18 patients had symptoms of active vasculitis at inclusion, while the 3 remaining patients with relapsing disease were included because of elevated inflammatory markers. All 18 patients had evidence of aortic FDG uptake of ≥2 at baseline PET/CT, and 8 patients (4 with newly diagnosed GCA and 4 with relapsing GCA) also had evidence of aortic dilation. Temporal artery biopsy was performed in 3 relapsing patients at disease onset, and it was positive in 2.

**Table 1.** Baseline characteristics of the patients

Study population (*n* = 18)	
Age, years	68.5 (10.6)
Sex	
Female	13 (72%)
Male	5 (28%)
Ethnic origin	
White	18 (100%)
Newly-diagnosed LV-GCA	9 (50%)
Relapsing LV-GCA	9 (50%)
Glucocorticoid pre-treatment	13 (72%)
CRP, mg/l	35 (36)
ESR, mm/h	56 (41)
Symptoms of active vasculitis	15 (83%)
Systemic symptoms	11 (61%)
PMR	7 (39%)
Signs or symptoms of vascular insufficiency^a^	4 (22%)
Cranial symptoms	1 (6%)
PETVAS	17.3 (5.1)
Aortic dilation	8 (44%)
Aortic diameters	
Ascending, mm	39.1 (4.4)
Descending, mm	31.1 (5.1)
Suprarenal, mm	26.6 (4.5)
Infrarenal, mm	21.7 (3.5)

Data are mean (s.d.) or *n* (%).

a At least one among upper extremity claudication, vascular bruits, abnormal radial pulse, upper extremity blood pressure discrepancy. LV-GCA: large vessel-GCA; PETVAS: PET vascular activity score.

All 18 patients received the three GC infusions and were started on s.c. TCZ, and 14 of the 18 patients completed the follow-up until week 52. Four patients dropped out before week 52: two before week 24 due to non-responsiveness to TCZ for inducing remission; one at week 24 due to relapse; one at week 29 due to withdrawal of informed consent (Fig. 1). Two patients discontinued TCZ at week 44 because of adverse events (cutaneous reaction and aortic aneurysm surgical repair), and both were followed up until week 52 without therapy. Of these two patients, one underwent PET/CT at week 52.

### Primary end points

Compared with the baseline value, a significant reduction in PETVAS was observed at weeks 24 and 52: mean change –8.6 and –10.4, *P* = 0.001 and 0.002, respectively (Tables 2–4).

**Table 2.** Clinical outcomes

Outcome	Week 24	Week 52
**Primary end points**		
Change in PETVAS compared with baseline, mean differences (95% CI)	–8.6 (–11.5 to –5.7)	–10.4 (–13.6 to –7.2)
*P*-value	0.001	0.002
Proportion of patients with relapse-free remission, *n* (%, 95% CI)	10/18 (56, 31–78)	8/17 (47, 23–72)
**Secondary end points**		
Proportion of patients with relapse-free clinical remission, *n* (%, 95% CI)	15/18 (83, 59–96)	13/17 (76, 50–93)
Proportion of patients with relapse-free EULAR remission, *n* (%, 95% CI)	13/18 (72, 47–90)	10/17 (59, 33–82)
Proportion of patients with new aortic dilation, *n* (%, 95% CI)	0	0
**End point of interest**		
Proportion of patients with progressive aortic damage compared with baseline, *n* (%, 95% CI)	3/16 (19, 4–46)	4/14 (29, 8–58)

PETVAS: PET vascular activity score.

**Table 3.** Imaging and laboratory parameter changes in the 16 patients at week 24

	Baseline	Week 24	Change from baseline to week 24 (95% CI)	*P*-value
PETVAS	18.3 (4.7)	9.6 (4.9)	–8.6 (–11.5 to –5.7)	0.001
ESR	56 (41)	4 (4)	–52 (–72 to –31)	<0.0001
CRP	35 (34)	1 (1)	–34 (–54 to –15)	0.001
Ascending	38.88 (3.26)	39.69 (4.42)	0.81 (–0.31–1.93)	0.141
Descending	31.50 (5.12)	32.31 (7.18)	0.81 (–0.39–2.02)	0.066
Suprarenal	26.88 (4.71)	27.56 (5.83)	0.69 (–0.18–1.55)	0.066
Infrarenal	21.19 (2.97)	21.50 (3.68)	0.31 (–0.23–0.85)	0.180

Data are mean (s.d.). PETVAS: PET vascular activity score.

**Table 4.** Imaging and laboratory parameter changes in the 14 patients at week 52^a^

	Baseline	Week 52	Change from baseline to week 52 (95% CI)	*P*-value
PETVAS	18.2 (4.3)	7.9 (4.6)	–10.4 (–13.6 to –7.2)	0.002
ESR	53 (45)	4 (3)	–49 (–79 to –19)	0.005
CRP	28 (30)	0.5 (0.3)	–28 (–47 to –1)	0.002
Ascending	38.69 (3.57)	39.85 (5.06)	1.15 (–0.34–2.65)	0.066
Descending	29.92 (2.18)	30.38 (2.50)	0.46 (0.06–0.86)	0.034
Suprarenal	25.46 (3.02)	25.85 (3.08)	0.39 (–0.14–0.91)	0.102
Infrarenal	20.31 (1.97)	20.46 (1.85)	0.15 (–0.07–0.38)	0.157

Data are mean (s.d.).

a 13 patients underwent PET/CT. PETVAS: PET vascular activity score.

The proportions of patients with relapse-free remission at weeks 24 and 52 were 56% (95% CI 31–78) and 47% (95% CI 23–72), respectively (Table 2). Despite the PETVAS reduction, 6 of the 16 PET/CTs (38%) were still considered active by the nuclear medicine physician’s interpretation at week 24 and 3 of 13 (23%) at week 52.

### Secondary end points

The proportions of patients with relapse-free clinical remission at weeks 24 and 52 were 83% (95% CI 59–96) and 76% (95% CI 50–93), respectively (Table 2). Fourteen (78%) of the 18 patients achieved clinical remission within 31 days, and 16 (89%) within 16 weeks. The mean time (s.d.) to clinical remission was 5.8 (3.9) weeks. The results remained similar when the 3 patients without symptoms at inclusion were excluded from the analysis (supplementary material available at *Rheumatology* online).

Following the EULAR consensus definitions for remission [16], the proportions of patients with relapse-free remission at weeks 24 and 52 were 72% (95% CI 47–90) and 59% (95% CI 33–82), respectively (Table 2).

At weeks 24 and 52, no patient showed new aortic dilation. Any change of ≥5 mm on serial CT was considered significant progression of aortic damage and was evaluated as an end point of interest (Table 2) [19]. Three dilated patients at baseline showed significant progression of aortic damage at week 24 (2 with newly diagnosed GCA and 1 with relapsing disease). Two of these 3 patients were in clinical remission and showed a significant reduction in PETVAS at week 24 (PETVAS change of –18 and –13, respectively). For one of these patients, surgical repair of an aortic aneurysm was required at week 44. Histopathology was not available. A fourth additional patient, dilated at baseline, showed a significant increase in aortic diameter at week 52. This patient, with newly diagnosed GCA, achieved relapse-free remission at week 24, showed significant reduction in PETVAS at weeks 24 and 52 (PETVAS change of –10 at week 24 and –14 at week 52), and was considered to be in clinical remission at week 52. All four patients with aortic dilation progression had evidence of active aortitis at baseline PET/CT.

Two (11%) of 18 patients did not respond to treatment, and rescue GC treatment was necessary. Of these two patients, one had persistent systemic symptoms (GC treatment was started at week 8) and the second had persistent PMR symptoms (GC treatment was started at week 15). Two patients (11%) relapsed (1 on week 24 and 1 on week 52), and GC treatment was started at the time of relapse. One patient, who achieved relapse-free remission at week 24 withdrew informed consent in week 29.

### Adverse events

TCZ and methylprednisolone pulse therapy were well tolerated, and no new or unexpected safety concerns were observed. One patient underwent aortic aneurysm surgical repair at week 44. No patient had vision loss or cerebrovascular events. All adverse events occurring during the study are reported in the supplementary material available at *Rheumatology* online.

## Discussion

In this prospective observational study (TOPAZIO), we confirmed that TCZ monotherapy after 3 days of pulses of methylprednisolone was safe and led to a significant reduction in vascular inflammation, as evaluated by PET/CT, at weeks 24 and 52. We selected vascular inflammation as the primary outcome measure, and we used PETVAS to quantify the arterial FDG uptake. We observed a mean significant change in PETVAS of –8.6 at week 24 and –10.4 at week 52.

In a prospective observational study, Quinn *et al.* observed that PETVAS was useful for assessing and monitoring vascular inflammation in patients with newly diagnosed or relapsing GCA treated with TCZ and GCs [20].

The RIGA study was an observational retrospective study assessing the change in vascular inflammation by using PETVAS in patients with new-onset, active LV-GCA under different treatments [21]. In the 19 patients treated with combined TCZ and GCs, the mean PETVAS change after a mean of 11.8 months was –11.7, very similar to that observed at 52 weeks in our patients treated with TCZ monotherapy (–10.4). These findings, indicating similar efficacy of the two treatment regimens in controlling vascular inflammation, suggest that GC exposure can be substantially reduced in GCA patients treated with TCZ.

Our co-primary end points were the proportions of patients with relapse-free remission at weeks 24 and 52. The definition of remission in LVV is still debated, and previous clinical trials have applied different definitions of remission as the end point, limiting the comparison of treatment effects [22]. Our definition of remission reflects our clinical practice, in which we routinely use repeated PET/CT scans in association with clinical manifestations, inflammatory markers, and progression of vascular damage to evaluate disease activity and treatment response. According to this definition, 56% of patients achieved relapse-free remission at week 24, and 47% at week 52. These results suggest that TCZ monotherapy after ultra-short-pulse GC not only controls the clinical symptoms of GCA and lowers acute phase responses, but also controls vascular inflammation in about half of the treated patients at 1 year.

In the two RCTs that showed the steroid-sparing effect and the efficacy of TCZ in reducing the relapse rate compared with placebo, remission was defined as the absence of flare and the normalization of the CRP [11, 12]. As TCZ suppresses the acute phase response, disease activity assessment in these two trials was mainly based on clinical signs and symptoms. To compare the results of our study with those of these two RCTs, we introduced as a secondary end point the rate of clinical remission, defined as the absence of any clinical signs and symptoms directly attributable to GCA, including normalization of CRP and ESR. According to this definition, 83% of patients were in clinical remission at week 24, and 72% at week 52, a proportion even higher than that seen in the GiACTA trial, in which 56% of the patients treated with TCZ weekly and 53% of those treated with TCZ every other week reached relapse-free remission at week 52 [11].

TOPAZIO is the first study exploring as an end point the EULAR definition of remission [16]: 72% of our patients achieved relapse-free remission at week 24, and 59% at week 52. However, 19% of patients considered in to be remission using the EULAR criteria at week 24, and 15% at week 52, had a PET/CT scan considered active by the nuclear medicine physician’s interpretation.

To date, the role of PET/CT in monitoring disease activity and predicting relapse remains unclear [23]. PET/CT was found useful in the assessment of LVV patients with poor clinical treatment response or suspicion of relapse after the reduction or the withdrawal of the therapy [24]. However, persistence of low-grade FDG vascular uptake has been reported in 50% or more of patients with LVV in remission after treatment [20, 23, 25]. It is unclear whether this persistent metabolic activity in the vascular wall represents subclinical vasculitis, remodelling, atherosclerosis, or a combination of these factors [18]. We believe that PET/CT provides important information on the control of vascular inflammation that complements clinical manifestations and inflammatory markers. Therefore, we decided to incorporate PET/CT in these novel multi-outcome domain remission criteria in GCA; this needs to be validated in larger studies.

The design of the present study is similar to that of the GUSTO trial, a proof-of-concept trial evaluating the efficacy and safety of TCZ monotherapy after ultra-short-term GC treatment in patients with new-onset GCA [14]. After 3 days of pulse therapy with methylprednisolone, followed by TCZ monotherapy, only 4 of the 18 enrolled patients (22%) met the primary end point (remission within 31 days and no relapse at week 24). The mean time to remission (defined as the disappearance of symptoms and normalization of CRP) was longer than anticipated in the protocol (11 weeks instead of 4 weeks). However, 78% of the 18 enrolled patients achieved remission within 24 weeks, and 72% remained relapse-free until 52 weeks. US of the temporal, axillary and subclavian arteries was performed at baseline, on days 2/3, and periodically until week 52. The intima-media thickness (IMT) showed a sharp decline after the GC pulses (day 2/3) in all evaluated arteries. However, the temporal arteries IMT increased to baseline levels at week 4, and then slowly decreased, paralleling the improvement of the clinical manifestations. The effect on the axillary and subclavian arteries was smaller and delayed, with 3 patients developing new lesions at week 4 [15].

In our study, the mean time to clinical remission was shorter than in the GUSTO trial (6 weeks), and 78% of patients achieved remission within 31 days. These differences were probably related to the different inclusion criteria used in the two studies. The GUSTO trial enrolled consecutive newly diagnosed GCA patients, the majority of whom had both cranial symptoms (ischaemic cranial manifestations were present in more than half of the patients) and aortitis on MRI (78%). Because we excluded patients with cranial ischaemic manifestations, the TOPAZIO study had more patients with systemic and fewer with cranial manifestations compared with the GUSTO trial (61% *vs* 33% and 6% *vs* 83%, respectively). The resolution of the cranial manifestations following TCZ monotherapy probably requires more time compared with the resolution of systemic manifestations, which are more related to a direct effect of IL-6.

In contrast to the GUSTO trial, in which a patient developed unilateral arteritic anterior ischaemic optic neuropathy 15 days after the third GC infusion, we did not observe visual manifestations in our patients. We believe that GCA patients without cranial ischaemic manifestations may represent a better subset for treatment with this regimen. However, because the TOPAZIO study did not include follow-up imaging before week 24, any intercurrent rebound of vessel wall inflammation may have been missed. Furthermore, we cannot exclude that the absence of continuous GC treatment may have contributed to the aortic dilation observed in 4 of our patients.

A third small prospective study from Japan confirmed the efficacy of TCZ monotherapy in GCA. In this study, 8 patients with newly diagnosed GCA were treated with TCZ monotherapy without GCs for 1 year. Of these, 75% of patients were in complete remission (defined as absence of vasculitic symptoms and normalization of CRP) at week 24, and this was maintained at week 52 [13].

We did not observe new aortic dilation at weeks 24 and 52, although the observational period was too short to evaluate this complication, which usually occurs 3–5 years after GCA diagnosis [26–28]. However, 4 patients (3 with newly diagnosed GCA and 1 with relapsing disease) with evidence of active aortitis and aortic dilation at baseline PET/CT examination showed progression of aortic damage (increase in aortic diameter of ≥5 mm) on serial CT, and 1 of these required surgical repair of aortic aneurysm. Three of the 4 patients were in clinical remission and had a significant reduction in PETVAS, while control PET/CT showed progression of aortic damage. It is unclear whether the progression of the aortic dilation was related to the persistence of smouldering subclinical vasculitis or represented evolving vascular damage unrelated to inflammation. Despite the reduction in vascular inflammation, TCZ monotherapy after ultra-short GCs was therefore not able to prevent progression of aortic damage at 1 year in half of the patients (4 out of 8) with baseline evidence of aortitis and aortic dilation. However, this subgroup by itself may represent a subset of patients with a higher risk of aortic dilation progression, independent of the type of treatment. Furthermore, the higher frequency of aortic dilation observed in the early phase of GCA in the present study (44%) compared with the previous study (up to 23% of patients) [8, 29, 30], may indicate the inclusion of patients with more severe aortic involvement. The question of whether TCZ is able to resolve vessel inflammation and prevent the development of vascular damage remains open; further studies with longer follow-up are needed.

Our study has several limitations: first, the observational design without blinded clinical assessment and a comparator group of patients treated with GC alone or with a different steroid-sparing agent; second, the small sample size, and third, the inclusion of patients from a single centre, which limits the generalizability of the results. The study strengths include the use of a standardized imaging protocol with a centralized reader to assess the response to treatment at different time points during the follow-up, and the homogeneously identified cohort of patients included with active LV-GCA. Furthermore, in contrast to the GUSTO trial [14], we enrolled both treatment-naïve and relapsing GCA; in this way the population of patients was less homogeneous, but more representative of the daily clinical practice. Finally, for the first time, we proposed multi-outcome domain response criteria that integrate clinical manifestations, laboratory markers and functional and morphologic imaging studies [22].

In conclusion, TCZ monotherapy after ultra-short-pulse GCs controlled the clinical symptoms of GCA and led to a significant reduction in vascular inflammation. However, it remains unknown whether TCZ is also able to prevent vascular damage. The TOPAZIO and GUSTO trial results suggest that there is still a high potential to further spare GCs when treating GCA with TCZ [14].

## Supplementary Material

kead215_Supplementary_Data

## Data Availability

Data are available on reasonable request. The individual anonymized data supporting the analyses contained in the manuscript will be made available on reasonable written request from researchers whose proposed use of the data for a specific purpose has been approved. Data will not be provided to requesters with potential or actual conflicts of interest, including individuals requesting access for commercial, competitive or legal purposes. Proposals and data access requests should be directed to the corresponding author. To gain access, data requestors will need to sign a data access agreement.
