# Supplementary material for: Treatment of giant cell arteritis with ultra-short glucocorticoids and tocilizumab: the role of imaging in a prospective observational study
Source: Rheumatology (Oxford). 2023 May 17;63(1):64–71. doi: 10.1093/rheumatology/kead215 (PMC10765153; doi:10.1093/rheumatology/kead215)
Supplement: kead215_Supplementary_Data [file kead215_supplementary_data.docx]

**Supplementary Material**

**Inclusion Criteria**

1. Age 50 years or more
2. PET/CT showing FDG uptake ≥2 in at least one large artery (aorta and its major branches) and considered consistent with active vasculitis by the evaluation of the nuclear medicine physician
3. At least one among
   1. ESR >40 mm/h or CRP >10 mg/l
   2. Cranial or systemic symptoms of GCA or symptoms of polymyalgia rheumatica (PMR).

**Exclusion Criteria**

1. Presence or history of ischemic cranial manifestations (jaw claudication, vision loss, amaurosis fugax, diplopia, stroke and transient ischemic attacks)
2. Treatment with more than 10 mg/day of prednisone (or equivalent) for more than 10 consecutive days in the previous three months
3. Rheumatic diseases (except for gout and CPPD/chondrocalcinosis) other than GCA or PMR
4. Chronic use of systemic GC with inability, in the opinion of the investigator, to withdraw GC treatment at day 3 according to protocol
5. Evidence of significant and/or uncontrolled concomitant disease such as, but not limited to, cardiovascular disease, nervous system, pulmonary, renal, hepatic, endocrine (in particular diabetes mellitus) or gastrointestinal disorders (including previously complicated diverticulitis) which, in the investigator’s opinion, would preclude patient participation or impact the benefit-risk ratio
6. Any condition or general state of health which, in the investigator’s opinion, would preclude participation in the study
7. Actual or recent myocardial infarction (within the last three months before the screening visit)
8. Significant cardiac disease (NYHA Class III and IV), known severe chronic obstructive pulmonary disease (COPD) (FEV1 < 50% predicted or Functional dyspnea > Grade 3 on the MRC Dyspnea Scale) or other significant pulmonary disease
9. Uncontrolled disease (such as asthma, psoriasis or inflammatory bowel disease) where flares are commonly treated with oral or injectable GCs
10. Known active infection of any kind, or any major episode of infection requiring hospitalization or treatment with i.v. anti-infectives within four weeks or completion of oral anti-infectives within two weeks before the screening visit
11. History of deep space/tissue infection (e.g. fasciitis, abscess, osteomyelitis) within 52 weeks before the screening visit
12. Any surgical procedure, including bone/joint surgery within eight weeks before the screening visit or planned within the duration of the study
13. History of recurrent severe or chronic infection
14. Lack of peripheral venous access
15. Body weight > 150 kg or BMI > 35
16. Previous treatment with tocilizumab
17. Previous treatment with any other biological agent within the last 6 months before the screening visit (12 months for rituximab)
18. History of severe allergic or anaphylactic reaction to any biological agent or known hypersensitivity to any component of tocilizumab
19. Receipt of any vaccine within 28 days prior to the screening visit
20. Positive tests for hepatitis B surface antigen (HBsAg) or hepatitis C serology
21. Positive Quantiferon-TB® test for latent tuberculosis (Tb) without subsequent INH prophylaxis
22. Patients with active Tb which had to be treated for Tb within 2 years before the screening visit
23. Absolute neutrophil count (ANC) < 2.0 x 103/µL, white blood cells < 2.5 x 103/µL, platelet count < 100,000/ µL
24. Hemoglobin < 8.0 g/dL
25. Concentrations of serum IgG and/or IgM below 5.0 mg/mL and 0.40 mg/mL, respectively
26. Serum creatinine > 2.0 mg/dL (200 µmol/L)
27. Alanine aminotransferase (ALT) or aspartate aminotransferase (AST) > 1.5 times the upper limit of normal (ULN)
28. Total bilirubin > 1.5 times the upper limit of normal (ULN)
29. Triglycerides > 400 mmol/dL (non-fasted) or > 250 mmol/dL (fasted) at screening
30. Evidence of malignant disease or malignancies diagnosed within the previous 5 years (except basal and squamous cell carcinoma of the skin or carcinoma in situ of the cervix uteri that have been excised and cured)

**PET/CT acquisition.**

Serum blood glucose was routinely checked and had to be <180mg/dl, while patients also fasted for at least 4h before i.v. injection of 2.5 MBq/kg ^18^F-FDG. Scans were acquired 1h after injection. All patients were in the supine position with their arms alongside the body. First a non-contrast low-radiation-dose CT scan followed by the PET scan from the base of skull to the knee was performed using a Discovery MI, GE hybrid 64 PET/CT machine (General Electric, Boston, Massachussets, USA).

**Secondary endpoints including only the 15 patients with symptoms at inclusion.**

The proportion of patients with relapse-free clinical remission at weeks 24 and 52 was 13 out of 15 (87%, 95% CI 60-98) and 12 out of 14 patients (86%, 95% CI 57-98), respectively (Table 2). Twelve (80%) of the 15 patients achieved clinical remission within 31 days. Mean time (SD) to clinical remission was 5.5 (3.4) weeks.

Following EULAR consensus definitions for remission, the proportion of patients with relapse-free remission at weeks 24 and 52 was 11 out of 15 (73%, 95% CI 45-92) and 9 out of 14 (64%, 95% CI 35-87), respectively.

**Adverse events**

Up to week 52, adverse events occurred in 10 patients. Neutropenia of less than 1.0×109/L occurred in 1 of 18 patients. TCZ was withheld in this patient until neutrophil count increased to more than 1.0×109/L, then it was administered every other week. Hypercholesterolemia requiring the start of statin therapy occurred in 2 patients. Two patients had cutaneous reaction, requiring discontinuation of TCZ at week 44 in one. Three of 18 patients had three infectious events (two COVID19 and one tooth infection). None of these infections was considered severe, neither required hospitalization. No patient had diverticulitis or a rise in transaminases more than five times the upper limit of normal. Methylprednisolone pulse therapy was well tolerated. No patient had severe cranial ischemic manifestations, particularly loss of vision related to arteritic anterior ischemic optic neuropathy or cerebrovascular events. One patient underwent aortic aneurysm surgical repair at week 44.
